# New Medical Journals

**Published:** 1851-12

**Authors:** 


*New Medical Journals.*— A new Medical Journal has been commenced in the city of New York, entitled the *Neu York Medical Times.* It is a monthly, of thirty-two pages, the terms being two dollars *per annum.*

The *New York Register* has been discontinued.

The *New Orleans Monthly Medical Register* is the title of another addition to the numerous periodicals now in existence in this country. This Journal contains twelve pages of reading matter, and the terms are one dollar *per annum.*

The field of medical periodical literature is ample, and, although the laborers are not few, there is abundance of room for more. We cordially extend the hand of fellowship to new co-workers, wishing them all the success and recompense that they may desire.

